# Thriving in Oxygen While Preventing ROS Overproduction: No Two Systems Are Created Equal

**DOI:** 10.3389/fphys.2022.874321

**Published:** 2022-04-04

**Authors:** O. Mendez-Romero, C. Ricardez-García, P. Castañeda-Tamez, N. Chiquete-Félix, S. Uribe-Carvajal

**Affiliations:** Departamento de Genética Molecular, Instituto de Fisiología Celular, Universidad Nacional Autónoma de México, Ciudad Universitaria, Mexico City, Mexico

**Keywords:** mitochondria, apoptosis, physiological uncoupling, ROS, oxygen avoidance, oxyregulators, oxyconformers

## Abstract

From 2.5 to 2.0 billion years ago, atmospheric oxygen concentration [O_2_] rose thousands of times, leading to the first mass extinction. Reactive Oxygen Species (ROS) produced by the non-catalyzed partial reduction of O_2_ were highly toxic eliminating many species. Survivors developed different strategies to cope with ROS toxicity. At the same time, using O_2_ as the final acceptor in respiratory chains increased ATP production manifold. Thus, both O_2_ and ROS were strong drivers of evolution, as species optimized aerobic metabolism while developing ROS-neutralizing mechanisms. The first line of defense is preventing ROS overproduction and two mechanisms were developed in parallel: 1) Physiological uncoupling systems (PUS), which increase the rate of electron fluxes in respiratory systems. 2) Avoidance of excess [O_2_]. However, it seems that as avoidance efficiency improved, PUSs became less efficient. PUS includes branched respiratory chains and proton sinks, which may be proton specific, the mitochondrial uncoupling proteins (UCPs) or unspecific, the mitochondrial permeability transition pore (PTP). High [O_2_] avoidance also involved different strategies: 1) Cell association, as in biofilms or in multi-cellularity allowed gas-permeable organisms (oxyconformers) from bacterial to arthropods to exclude O_2._ 2) Motility, to migrate from hypoxic niches. 3) Oxyregulator organisms: as early as in fish, and O_2_-impermeable epithelium excluded all gases and only exact amounts entered through specialized respiratory systems. Here we follow the parallel evolution of PUS and O_2_-avoidance, PUS became less critical and lost efficiency. In regard, to proton sinks, there is fewer evidence on their evolution, although UCPs have indeed drifted in function while in some species it is not clear whether PTPs exist.

## Introduction

During the great oxygenation event (GOE), 2.5 to 2 billion years ago, oxygen concentration ([O_2_]) increased thousands of times, leading to the first massive extinction (Lane, 2002; Reinhard et al., 2016). Except for those living in anaerobic niches, surviving organisms had to adapt and thus aerobic metabolism developed: organisms harness the highly exergonic O_2-_reduction, increasing ATP yields up to 16 times. However, the toxicity of the highly motile free radicals of O_2_, the ROS had to be dealt with, and many strategies were developed (Weiss et al., 2016). Detoxifying enzymes such as superoxide dismutase, catalase and the glutathione peroxidase system eliminate ROS (Cortassa et al., 2014). These iron-containing antioxidant enzymes are present in strict anaerobes, possibly inherited from their anaerobic prokaryotic ancestors (Ślesak et al., 2016).

Another mechanism against oxygen toxicity is to prevent the non-catalyzed partial reduction of O_2_ through physiological uncoupling systems (PUS), which increases the rate of electrons in flow in respiratory chains (RC) lowering both cellular [O_2_] and free radical concentrations (Kadenbach, 2003; Guerrero-Castillo et al., 2011; Uribe-Carvajal et al., 2011; Guerrero-Castillo et al., 2012; Cabrera-Orefice et al., 2015). PUS may be intrinsic, as branched RCs or extrinsic, and as in proton sinks. Branched RCs are those that contain non-proton pumping redox enzymes, while mitochondrial proton sinks include uncoupling proteins (UCPs) and permeability transition pores (PTP) (Kadenbach, 2003; Demine et al., 2019; Zhao et al., 2019).

**FIGURE 1:**
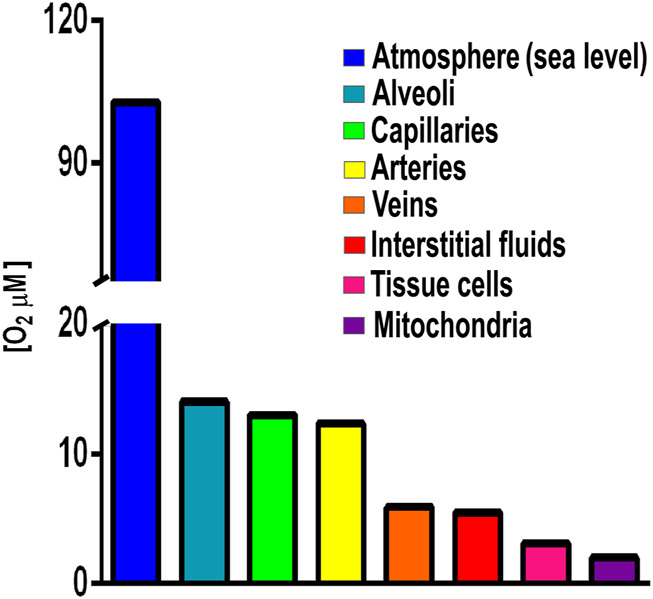
Oxygen concentrations in different compartments in an oxyregulator organism. Atmospheric [O_2_] 1,028 μM, lung alveoli [O_2_] 143 μM; capillaries [O_2_] 130 mM; arteries [O_2_] 123 μM; interstitial fluids [O_2_] 55 μM; tissue cells [O_2_] 31 μM; and mitochondria [O_2_] 20 µM. Values from Rosas-Lemus et al., 2016 and references therein.

Initially, for many species anaerobic niches must have been the only option for survival (Müller et al., 2012). Total avoidance of O_2_ is observed in anaerobic organisms such as those living in animal digestive tubes (Fenchel and Finlay, 1994). However, when accidentally exposed to high [O_2_], anaerobic organisms may differentially express branched RCs (Rosas-Lemus et al., 2016; Jayawardhane et al., 2020). Another avoidance strategy is association into biofilms, where surface cells limit O_2_ diffusion, creating an anaerobic internal microenvironment (Stewart, 2003). Perhaps these structures became stable, giving birth to multicellular organisms that move, migrating to areas where [O_2_] is lower (Abele et al., 2007). Among pluricellular organisms, fungi and arthropods are oxyconformers, i.e., their control of O_2_ diffusion is partial (Martínez-Cruz et al., 2012). The most sophisticated O_2_ avoidance system first appeared in fish and evolved in amphibians, reptiles, birds, and mammals, which developed a gas-impermeable epithelium plus a specialized external respiratory system (gills or lungs) (Stamati et al., 2011). These are the oxyregulator organisms where [O_2_] reaching internal cells is 20–31 μM or four to five hundred times less that atmospheric [O_2_] which is 1,026 μM at sea level (Figure 1) (Rosas-Lemus et al., 2016). Interestingly, amphibians already have lungs, even when their skin is still permeable to gases and participates in O_2_/CO_2_ exchange (skin breathing) (Tattersall, 2007).

During evolution from prokaryotes to unicellular eukaryotes to pluricellular organisms, the ability to exclude oxygen improved while in contrast, respiratory chain branching was progressively lost (Hsia et al., 2013). Increasing O_2_-avoidance efficiency rendered alternative redox enzymes superfluous and thus these were lost (McDonald et al., 2009). However, O_2_ overexposure accidents are more dangerous when PUS become less efficient (Rosas-Lemus et al., 2016).

The amount of O_2_ consumed by mitochondria is determined by energy demand rather than availability; at high [ATP], O_2_ pressure is higher than 0.5 mm Hg^+^, while addition of an uncoupler (CCCP or FCCP) increases the respiratory rate 6–10 times while lowering pO_2_ to less than 0.03 mm Hg^+^ (Gnaiger et al., 1995). When cells are treated with an uncoupler, the decrease in pO_2_ is significantly smaller than in isolated mitochondria, suggesting that there are intracellular diffusion barriers in the cytoplasm (Wilson, 1990). The diffusion barriers are probably more difficult to estimate in whole tissues and organisms (Slggaard-Andersen et al., 1995). In mammals, the mitochondrial pO_2_ at state 3 only slightly lower than at state 4, while adding an uncoupler drives pO_2_ levels to dangerously low levels (Rumsey et al., 1990; Guidot et al., 1995; Steinlechner-Maran et al., 1996). This illustrates the intricate interaction of diffusion gradients, metabolic states, and coupling/uncoupling states on respiratory metabolism in oxyregulator organisms. In contrast, *Saccharomyces cerevisiae* cells collected during log phase growth in lactate it exhibits a superoxide scavenging activity that directly correlates with oxygen tension (Gnaiger et al., 1995). Thus, mitochondria from oxyregulators are probably more sensitive to hypoxia and reoxygenation than shrimp or yeast; let alone bacteria (Martínez-Cruz et al., 2017).

Here, we analyze the parallel evolution of PUS and avoidance strategies. Special attention is given to the branched structure in RCs. It is observed that as species developed better avoidance mechanisms, branching of their respiratory chains decreased, probably due to an effort to become more efficient to produce ATP and to the decreased need to control ROS overproduction in an O_2_-controlled environment. Whether this decrease in resistance to sudden oxygenation applies to proton sinks is not as well defined. Nonetheless, some considerations are made at the end of the chapter.

## Prokaryotes

When GOE arrived, most organisms were prokaryotes and the first eukaryotes were only beginning (Shikama and Matsuoka, 2004). All anaerobic species had to either avoid the toxic high [O_2_] environment or develop the ability to deplete O_2_ adapting their respiratory chains (RC) (Pahl and Baeuerle, 1994). RC constituents are at least substrate dehydrogenases that transferring electrons to a quinone pool and terminal reductases receiving electrons from quinones. Substrate-specific dehydrogenases transfer reducing equivalents from various donor substrates (NADH, succinate, glycerol phosphate, formate, hydrogen, pyruvate, and lactate) to a quinone pool (menaquinone, ubiquinone or dimethylmenaquinone) (Unden and Bongaerts, 1997). Then terminal reductases transfer electrons from quinol to a final electron acceptor (Brunori et al., 2005). In aerobic metabolism the terminal electron acceptor is O_2_ and thus reductases are termed oxidases (Cotter et al., 1990). As an additional element, some bacteria use quinol to reduce cytochrome *c* (Borisov and Verkhovsky, 2015).

Facultative bacteria express a highly adaptable RC allowing them to live in different environments, e.g., *Escherichia coli* lives in highly aerobic to anaerobic environments through the differential expression of different RC redox enzymes (Borisov and Verkhovsky, 2015). Differential expression of redox enzymes seems to protect the cell against stress, e.g. cytochrome *bd-I* has been reported to protect *E. coli* against H_2_O_2_ toxicity (Borisov et al., 2021). *Staphylococcus epidermidis* is a facultative anaerobe living in a broad range of [O_2_], including human skin, where [O_2_] varies from 2 to 5% (Peyssonnaux et al., 2008), ischemic/anoxic tumors, and abscesses, where [O_2_] is zero (Atkuri et al., 2007; Wiese et al., 2012). At different [O_2_], *S. epidermidis* RC composition varies widely (Table 1). In high O_2_, *S. epidermidis* expresses five oxido-reductases, namely glycerol-3-phosphate dehydrogenase, pyruvate dehydrogenase, ethanol dehydrogenase, and succinate dehydrogenase, as well as the cytochromes *bo* and *aa3*. Under these conditions biofilms production is minimal (Uribe-Alvarez et al., 2016). At low [O_2_], pyruvate dehydrogenase and ethanol dehydrogenase levels drop by 50%, while glycerol-3-phosphate dehydrogenase and succinate dehydrogenase levels vanish; among reductases, cytochrome *bo* increases, cytochrome *aa3* disappears, and nitrate reductase activity is present. Under anaerobic conditions, quinone donors do not vary further, while cytochrome *bo* decreases, and nitrate reductase is the predominant terminal electron acceptor (Uribe-Alvarez et al., 2016). Again, differential redox enzyme expression seems to protect *S. epidermidis* against high O_2_ concentrations. This response is coordinated with biofilm generation (see below) (Fang et al., 2016; Pedroza-Dávila et al., 2020).

**TABLE 1:** Enzyme expression at different [O_2_] (High Low or Zero) in the *S. epidermidis* branched respiratory chain.

Metaquinone e^−^ Donors	[O_2_]		
	H	L	Z
Type-2 NADH Dehydrogenase (NDi2)	+	+	+
Menaquinone Oxidase Complex (MQO)	+	+	+
Lactate Dehydrogenase (LDH)	+	+	+
Glycerol-3-Phosphate Dehydrogenase (GDH)	+	−	−
Succinate Dehydrogenase (SDH)	+	−	−
Alcohol Dehydrogenase (ADH)	+	+	+
Pyruvate Dehydrogenase (PDH)	+	+	+
Metaquinone e^-^ Acceptors			
Cytochrome *bo* Oxidase	+++	++	+
Cytochrome *aa3* Oxidase	+	−	−
Nitrate Reductase	−	+	+++

Cells were grown at High, Low or Zero [O_2_]. Under these conditions, some enzymes were expressed (+) or not (−), while others, namely Cytochrome bo Oxidase and Nitrate Reductase exhibited different expression levels (not expressed or increasingly expressed, +. to +++) (Data taken from Uribe-Alvarez et al., 2016).

Another interesting facultative bacterium is *Bacillus cereus,* which during sporulation, dormancy, or germination expresses different RC activities depending on its menaquinone concentration (Escamilla et al., 1988). NADH oxidase activity is inactivated during sporulation stages III to VI, most likely due to a substantial drop in menaquinone. During the same time frame, NADH oxidase in the mother cell progressively decreases to about 50%. Menadione restores NADH-dependent respiration and cytochrome reduction in dormant spore membranes (Escamilla et al., 1988). *Mycobacterium tuberculosis* is an obligate aerobe with a branched respiratory chain where electrons may travel from the cytochrome *bc1* complex to an *aa3*-type cytochrome *c* oxidase or may enter directly to a cytochrome *bd*-type quinol oxidase. Overexpression of cytochrome *bd* is linked to enhanced peroxide resistance, so this may be a survival strategy against the host immunological response (Lu et al., 2015).

Cytochrome *cbb3*, together with the *bd* oxidase, plays a key role in the protection of O_2_-sensitive nitrogenase in *Azorhizobium caulinodans*, a bacterium that develops a nitrogen-fixing symbiosis with plants of the genus *Sesbania*. Bacteria with *bd*-type oxidases have been found to be resistant to nitric oxide (NO), peroxynitrite, sulfide, ammonia, and cyanide. This is most likely why harmful bacteria have so much cytochrome *bd*. Because these enzymes are not found in eukaryotes, they are particularly appealing as prospective targets for novel antibacterial drugs (Borisov et al., 2021). One last example is *Neisseria gonorrhoeae*, which needs nitrite reductase, cytochrome *c* peroxidase and nitric oxide reductase to survive under stress and thus enhance its pathogenicity (Apicella et al., 2011; Phillips et al., 2012).

*Rickettsia prowazekii* (McGinn and Lamason, 2021), *Wolbachia sp*., and *Sodalis* (Attardo et al., 2020) are obligate endosymbiont bacteria that reside inside the cells of their hosts. D'Autréaux and Toledano (2007) speculate that these organisms invade the cytoplasm to live in a microaerophilic environment with O_2_ consuming organelles and ROS detoxifying enzymes. It is unclear if obligatory endosymbionts have a respiratory chain that can help host mitochondria deplete intracellular oxygen as even in the stationary phase*,* may force the host to maintain a high respiratory activity (Uribe-Alvarez et al., 2019).

## Unicellular Eukaryotes

Unicellular organisms show high adaptability to a wide range of [O_2_] in the medium through manipulation of their own anaerobic/aerobic metabolism (Bunn and Poyton, 1996; Johnson, 2019; López-Barneo and Simon, 2020), using detoxifying enzymes (Sies, 1993; Inupakutika et al., 2016; Lyall et al., 2020) or uncoupling proteins (UCPs) (Jarmuszkiewicz et al., 2010; Busiello et al., 2015; Demine et al., 2019; Nicholls, 2021).

**FIGURE 2:**
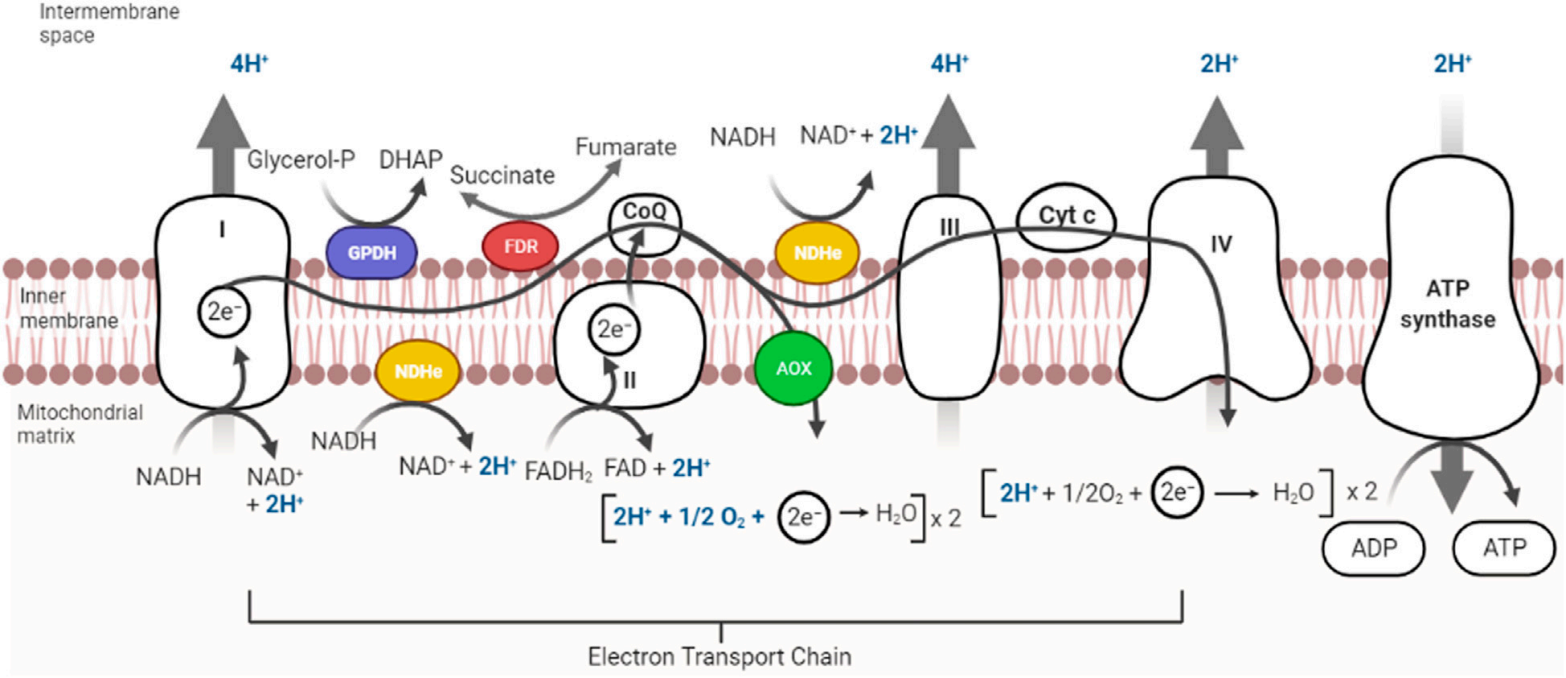
Components in mitochondrial respiratory chains. Most mitochondrial RCs are constituted by the classic complexes I, II, III, and IV. In addition, some RCs contain alternative redox enzymes donating electrons to the quinone pool such as alternative NADH dehydrogenase (in yellow), Glycerol-P-dehydrogenase (purple) or fumarate reductase (red). An alternative oxidase (AOX) oxidizes quinol reducing O_2_. Branched RCs skip proton pumps, decreasing proton pumping stoichiometry. Complex V uses proton gradients to produce ATP.

The ability to sense and respond to variations in [O_2_] is essential for survival (Cadenas, 1989). In eukaryotic cells ROS are produced in the reactions catalyzed by NAD(P)H oxidase and by some other specialized oxidases and as a byproduct of many redox reactions (Turpaev, 2002). Oxyconformer organisms (see below) have branched RCs (McDonald et al., 2009). The classical mitochondrial oxidative phosphorylation (OxPhos) components in the mitochondrial inner membrane are four respiratory complexes plus a fifth complex, the ATP synthase (Unden and Bongaerts, 1997). In eukaryotic cells RC branching is not as varied as in prokaryotes. The branches found are type-2 NADH dehydrogenases (NDH2) and alternative oxidases (AOX) (Figure 2) (Saari et al., 2019).

NDH2 reduces ubiquinone as does Complex I. However, NDH2 does not pump protons, it is not inhibited by rotenone, and it is not a transmembrane protein (Rasmusson et al., 2004; McDonald and Gospodaryov, 2019). NDH2 has been found in a variety of organisms, including plants, fungi, and yeasts. In fungi, these enzymes are found on both sides of the mitochondrial membrane, allowing them to oxidize either cytosolic or mitochondrial NAD(P)H. For example, *S. cerevisiae* has two external NDH2s (Nde1 and Nde2) plus one internal NDH2 (Ndi1) (Yamashita et al., 2018). In *S. cerevisiae* and *Saccharomyces carlsbergensis* Complex I is substituted by three alternative NDH2s (Helmerhorst et al., 2002). NDH2s may be in either side of the mitochondrial inner membrane, oxidizing NADH from the matrix or from the cytoplasm (Rigoulet et al., 2004).

The ultimate physiological uncoupling effect occurs when NADH dehydrogenases act in concert with AOX (Kerscher et al., 2002). AOX reduces O_2_ substituting the cytochrome pathway (Joseph-Horne et al., 2001). Alternative redox enzymes are induced during stress or in the stationary phase (Medenstsev et al., 1999; Medenstsev et al., 2002). At high [O_2_], AOX may decrease ROS production. The different O_2_ affinities of the cytochrome (K_m_ = 0.1 µmol) and alternative (K_m_ = 10–20 µmol) pathways enable COX to sustain OxPhos while AOX lowers [O_2_] (Gupta et al., 2015). These enzymes may also coexist with the usual complexes as in *Yarrowia lipolytica*, where an external NDH2 (Nde) and an AOX coexist with Complexes I to IV (Kerscher et al., 1999; Kerscher et al., 2002; Guerrero-Castillo et al., 2009). In *Candida parapsilosis* and *Candida albicans* antibiotic resistance has been linked to AOX expression (Guérin and Camougrand, 1986; Milani et al., 2001; Ruy et al., 2006). Additionally, in *C. albicans* the branched respiratory chain and UCP are important for decreasing ROS and modulating cell proliferation (Jarmuszkiewicz et al., 2000; Ruy et al., 2006).

In *Y. lipolytica* during the logarithmic growth phase, Nde interacts with complexes III–IV both of which are proton pumps (Guerrero-Castillo et al., 2009). In contrast, during the stationary growth phase, electrons are directly transferred from alternative NDH2 to AOX, thus uncoupling oxidative phosphorylation and decreasing ROS production (Guerrero-Castillo et al., 2012). *Debaryomyces hansenii* is a halotolerant yeast with a branched respiratory chain constituted by complexes I to IV plus an Nd2e and an AOX. In the stationary phase, a permeability transition pore opens and matrix NADH is depleted, which inactivates Complex I; then the alternative pathway becomes important for O_2_ depletion and prevention of ROS overproduction (Cabrera-Orefice et al., 2014). In *D. hansenni* grown in the presence of high osmolyte concentrations, AOX is coupled to complex I, promoting proton pumping even under conditions where the cytochrome pathway is compromised (Figure 2) (Garcia-Neto et al., 2017).

## Biofilms

**FIGURE 3:**
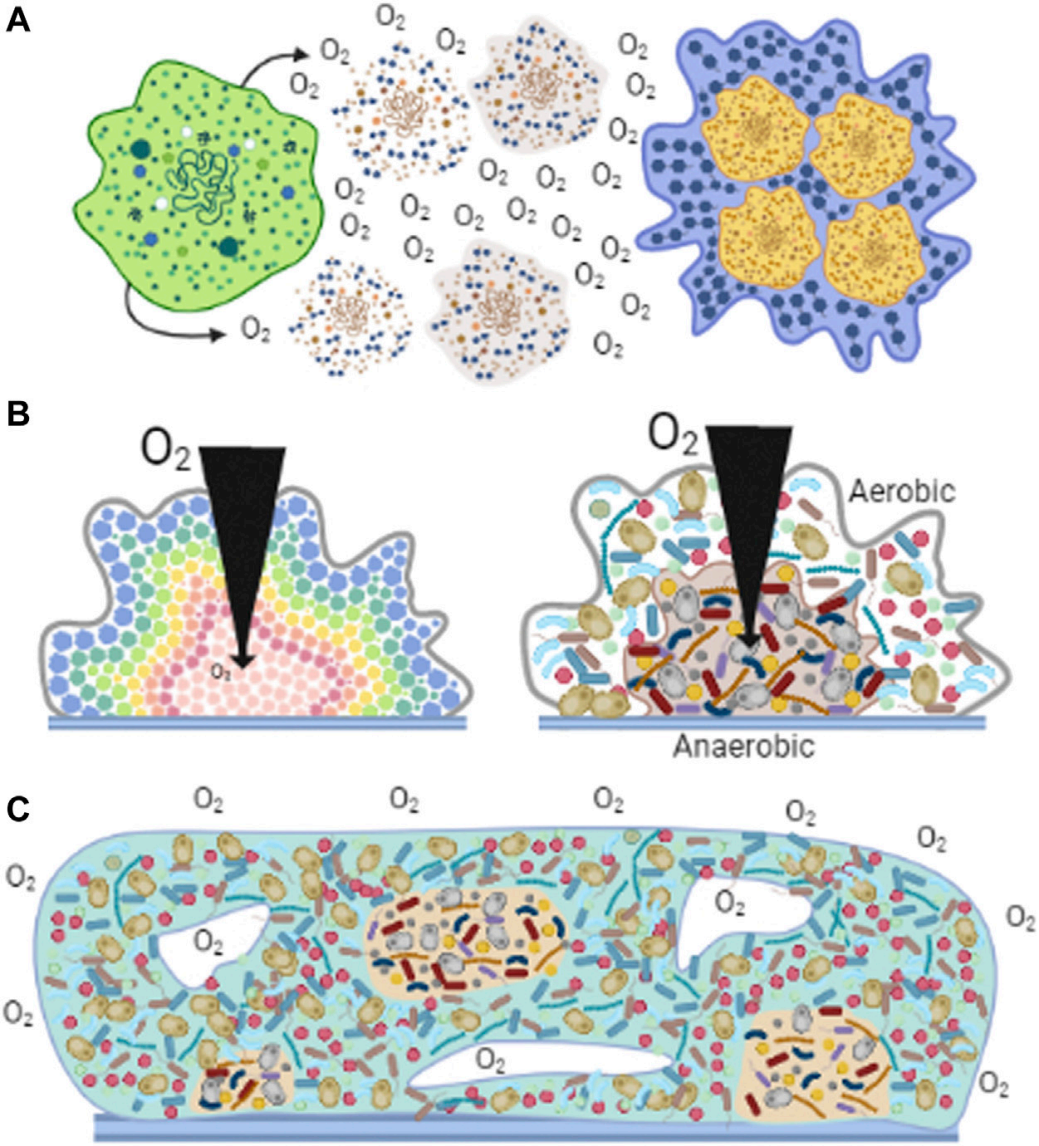
Biofilms observed in nature. Cells associate to form biofilms forming different structures. **(A)** Cells react to high [O_2_] by excreting polysaccharides that help them aggregate (Wingender et al., 1999). **(B)** Diversity in biofilms. In homogeneous aggregates (left panel), at different biofilm depths, cells perceive different levels of O_2_, nutrients and other metabolites and thus adapt their metabolism (López et al., 2010). In heterogeneous aggregates (right panel), aerobic organisms are located on the surface, using O_2_ before it enters deeper parts of the aggregate, enabling hypo/anaerobic organism to grow (Vilne et al., 2021). **(C)** Generation of microenvironments. Diverse cell species distribute according to their affinities for nutrients or [O_2_] (Wessel et al., 2014). The formation of channels (white spaces) allows the dynamic distribution of molecules and providing a path for waste disposal (Frances et al., 2001).

Biofilm formation was developed soon after the beginning of life (Westall et al., 2000). Organisms became capable of anchoring themselves near a feeding source or away from a toxic substance (Davey and O’toole, 2000; Azúa-Bustos et al., 2009; Wessel et al., 2014; Hall and Mah, 2017). Biofilms regulate exposure of individuals in the community to outside factors, where nutrients, oxygen, or molecules have a specific diffusion rate (Costerton et al., 2003; Stewart, 2003; Harrison et al., 2005). Aggregated cells were held together by secreted polysaccharides (Wingender et al., 1999). The carboxyl and phosphoryl groups in these polysaccharides led to biofilm mineralization and eventual fossilization (Westall et al., 2000). Biofilm fossils have provided morphological evidence suggesting that more than one microorganism species lived in these communities (Frances et al., 2001). Biofilms have been preserved over time as one of the most effective mechanisms of protection against many factors, in addition to varying [O_2_] (Figure 3A). It is currently preserved in most unicellular organisms such as bacteria, archaea, algae, some fungi, and protozoa (De la Fuente-Núñez et al., 2013).

Biofilms provide favorable microenvironments enhancing the development of organisms (Wessel et al., 2014). In biofilms formed by a single species, cell metabolism may vary depending on their location in the agglomerate, resulting in communities with metabolically active and inactive cells, or cells expressing different genes (Figure 3B) (López et al., 2010). i.e., in *S. epidermidis* changing [O_2_] affects growth rate, oxygen consumption, ATP synthesis and ROS resistance by expressing a defective respiratory chain and impact in the formation of biofilm (Pedroza-Dávila et al., 2020).

Low [O_2_] increases the synthesis of biofilm-associated compounds, including the cell adhesion-promoting extracellular polysaccharide -1,6-linked glycosaminoglycan (Cramton et al., 1999). Thus, biofilms would allow cells to grow in hypoxic environments. Still, at high [O_2_], biofilms formed by *Shewanella putrefaciens*, are regulated by [O_2_] (Wu et al., 2013). *S. putrefaciens* has an oxygen-sensitive diguanylate cyclase that regulates the expression of an BpfA adhesin and allows biofilms to be formed at high [O_2_] (Wu et al., 2013). In addition to their regulation of BpfA, diguanylate cyclases modulate the abundance of cyclic di-3′,5′-guanylate, a second messenger that regulates many bacterial behaviors, such as motility and biofilm formation (Valentini and Filloux, 2016).

In biofilms formed under anaerobic or hypoxic conditions, such as in *C. albicans*, biofilm regulators in normoxia such as hyphae promoters and glycosyltransferases do not interfere with biofilm development in anaerobic conditions (Rossignol et al., 2009; Stichternoth and Ernst, 2009), which means that the requirements for biofilm formation in hypoxic environments are different from those present under oxygenated conditions. In the anaerobic hyperthermophiles *Thermotoga maritimos*, *Archaeoglobus fulgidus*, and *Methanococcus jannaschii*, biofilm formation can be induced by high pH or ultraviolet light in addition to high [O_2_] (Pysz et al., 2004).

Besides the mentioned mutual protection against the environment, biofilms led to greater socialization (information exchange), enhancing survival abilities in participating species (Penesyan et al., 2021). In this regard, it has been proposed that biofilms led to the formation of eukaryotic cells: the “third space hypothesis” suggests some cells in the aggregates eventually stopped working for the common good, becoming predators and then, probably through phagocytosis incorporated future mitochondria (Davey and O’toole, 2000). Another hypothesis suggests that eukaryotic cells evolved through the exchange of “hyperstructures” between multiple types of cells, forming a “metacell”, which over time became integrated, compacted, and simplified to what we now know as eukaryotic cells (Norris and Root-Bernstein, 2009; Bateman, 2020).

Different species may associate in biofilms profiting from [O_2_] gradients or using secondary metabolite concentration gradients to find a niche within the community (Bradshaw et al., 1996; Penesyan et al., 2021). An interesting case is a biofilm found in the hot water pipes of residential buildings in the city of Riga (Latvia), where a strictly anaerobic microorganism (*Thermodesulfovibrio*) and a strictly aerobic species (*Phenylobacterium*) coexisted (Vilne et al., 2021). In this biofilm the aerobic organism was located mostly on the outer surface, while the anaerobic organism was inside the biofilm (Fox et al., 2014). In addition, biofilms can exist within other organisms; a clear example of this is the bovine digestive system where bacteria, protozoa and yeasts are embedded in the large biofilm covering the mucosal layer of the rumen (Harrison et al., 2005).

Biofilms formed by heterogeneous organisms show some similarities with multicellular organisms (Penesyan et al., 2021). The microenvironment generated by diffusion and distribution of compounds promotes different functions for different organisms much as in multicellular organisms (Penesyan et al., 2021). Thus, cells in the circumference of the biofilm deliver nutrients to the deeper zones. In addition, surface cells may control O_2_ distribution to the inner cells (de Beer et al., 1994). Ducts and channels resembling the circulatory systems in multicellular organisms are also found in biofilms and likely participate in the distribution of nutrients or O_2_ as well as in the outflow of wastes (Frances et al., 2001). Still, in biofilms microorganisms can survive independently, an ability that was lost in the specialized cells from multicellular organisms (Figure 3C) (Penesyan et al., 2021).

## Oxyconformer Pluricellular Organisms

Early in evolution, the control of O_2_ diffusion into pluricellular organisms was only partial, so organisms had to deal with sudden changes in [O_2_]. These organisms are termed oxyconformers (van Winkle and Mangum, 1975). Among these the phylum Arthropoda is the most numerous oxyconformer on the planet (Martínez-Cruz et al., 2012; Stork, 2018). Crustaceans are aquatic oxyconformers who confront a circadian cycle of water O_2_ levels throughout their lifetimes. To avoid overproducing ROS, crustaceans exhibit different strategies. The ghost shrimp *Lepidophtalmus louisianensis* and the branchiopod *Artemia franciscana* can survive in severely hypoxic and even anoxic settings by slowing down their respiratory and metabolic rates (Martínez-Cruz et al., 2017). When ambient oxygen levels are low, the white shrimp *Litopenaeus vannamei* can slow down its metabolic rate controlling mitochondrial function (Jiménez-Gutiérrez et al., 2014).

As in unicellular eukaryotes, alternative oxidase (AOX) is present in hypoxia-tolerant invertebrates from the phylum Porifera, Cnidaria, Nematoda, Annelida, Mollusca, and Echinodermata (McDonald and Gospodaryov, 2019). In these organisms AOX expression is enhanced under stress or hypoxia, e.g., in *Urechis unicinctus* the levels of AOX mRNA increase under stress (McDonald and Vanlerberghe 2004; McDonald et al., 2009).

AOXA and AOXB are two AOX variants found in abundance in the eastern oyster *Crassostrea virginica.* Under normal settings, AOXA has greater expression than AOXB, but under hypoxia, AOXB is the dominant form, and suggesting that it might be a stress-adaptation (Liu and Guo, 2017). Under anoxia, *A. franciscana* cysts may remain for years as a cyst and survive hatching in highly oxygenated water probably aided by AOX activity (Clegg and Conte, 1980; Rodriguez-Armenta et al., 2018). In the bivalve *Arctica islandica*, when OxPhos activity decreases, AOX helps maintain the mitochondrial respiratory rate probably aiding in prevention of ROS overproduction (Abele et al., 2007). In the Pacific giant oyster *Crassostrea gigas*, a cycle of hypoxia/re-oxygenation leads to a large increase in AOX expression (Sussarellu et al., 2012).

A mechanism complementing the differential expression of RC alternative redox enzymes, motile aquatic organisms such as *L. vannamei* and *Artemia sp* migrate, both during the day or in different stages of growth to those regions in water where they find optimal [O_2_] for their metabolism (Rosas-Lemus et al., 2016).

## Oxyregulators

Beginning with fish, organisms were enveloped in a gas impermeable epithelium that excluded O_2_. At the same time, specialized organs were developed which delivered exact quantities of O_2_ to internal tissues. Internalized O_2_ was not free, but tightly bound to different proteins such as hemoglobin or myoglobin (Bonaventura and Bonaventura, 1980; Reeder and Wilson, 2005). Through this mechanism, organisms are highly efficient to avoid the effect of ambient [O_2_] variations (Reinhard et al., 2016; Rosas-Lemus et al., 2016; Leiva et al., 2018). These organisms are termed oxyregulators and possess an optimal O_2_-avoidance system (Prosser, 1955). However, this led to an unwanted consequence as physiological uncoupling mechanisms became obsolete and RC branching disappeared and thus any accidental variation within the organism was highly dangerous. In oxyregulators, RCs lost all alternative redox enzymes, optimizing OxPhos. However, cells became more susceptible to variations in [O₂]. Still, in eukaryotes other PUS, and the proton sinks. It is not clear to what extent these can prevent cell death in oxyregulators.

## Extrinsic Physiological Uncoupling Systems (Proton Sinks)

In addition to RC branching, extrinsic mechanisms PUS were developed in eukaryotes. The uncoupling proteins (UCPs) and the permeability transition pore (PTP) are both immersed in the inner mitochondrial membrane (Guerrero-Castillo et al., 2011).

UCPs belong to the six-transmembrane helix family of mitochondrial transporters (Palmieri, 2014). UCPs help minimize ROS generation by partially dissipating the proton electrochemical gradient (Luévano-Martínez, 2012; Berry et al., 2018; Ji et al., 2020). In general, UCPs are needed for protection against stress, although it seems that their functions have drifted in diverse organism and these have been linked to the control of immunity as well as a variety of diseases such as sepsis, diabetes, and cancer (Dutra et al., 2018; Tian et al., 2018; Ding et al., 2019). Proton and chloride transport across lipid bilayers can be catalyzed by UCPs, suggesting that they play a role in decreasing ROS production through OxPhos uncoupling (Echtay et al., 2018; Gaudry and Jastroch, 2019).

Mitochondrial UCP activity is induced by fatty acids and inhibited by purine nucleotides (Lunetti et al., 2022). In insects and crustaceans, the distribution of UCP varies by species, tissues/organs, and developmental stage (Slocinska et al., 2011; Slocinska et al., 2012; Alves-Bezerra et al., 2014; Da-Ré et al., 2014; Mendez-Romero et al., 2020). Different roles have been proposed for mitochondrial UCPs in invertebrates, e.g., in the *Drosophila melanogaster*, DmUCP5 is involved in metabolic homeostasis (Sánchez-Blanco et al., 2006), while in the cold, at 15°C DmUCP4C is necessary for larvae transition to adulthood, suggesting that uncoupling respiration allows the fly to be cold-tolerant (Da-Ré et al., 2014) and DmUCP4 export aspartate from mitochondria (Lunetti et al., 2022). In the worm *Caenorhabditis elegans,* UCP4 confers cold tolerance (Iser et al., 2005), and transport succinate (Pfeiffer et al., 2011). In all, the main physiological role of UCPs may be protection against mitochondrial oxidative stress, as demonstrated in mitochondria from the cockroach *Gromphadorhina coquereliana* (Slocinska et al., 2011), the beetle *Zophobas atratus* (Slocinska et al., 2013), the blood-sucking bug *Rhodnius prolixus* (Alves-Bezerra et al., 2014) and the white shrimp *Litopenaeus vannamei* (Mendez-Romero et al., 2020).

**FIGURE 4:**
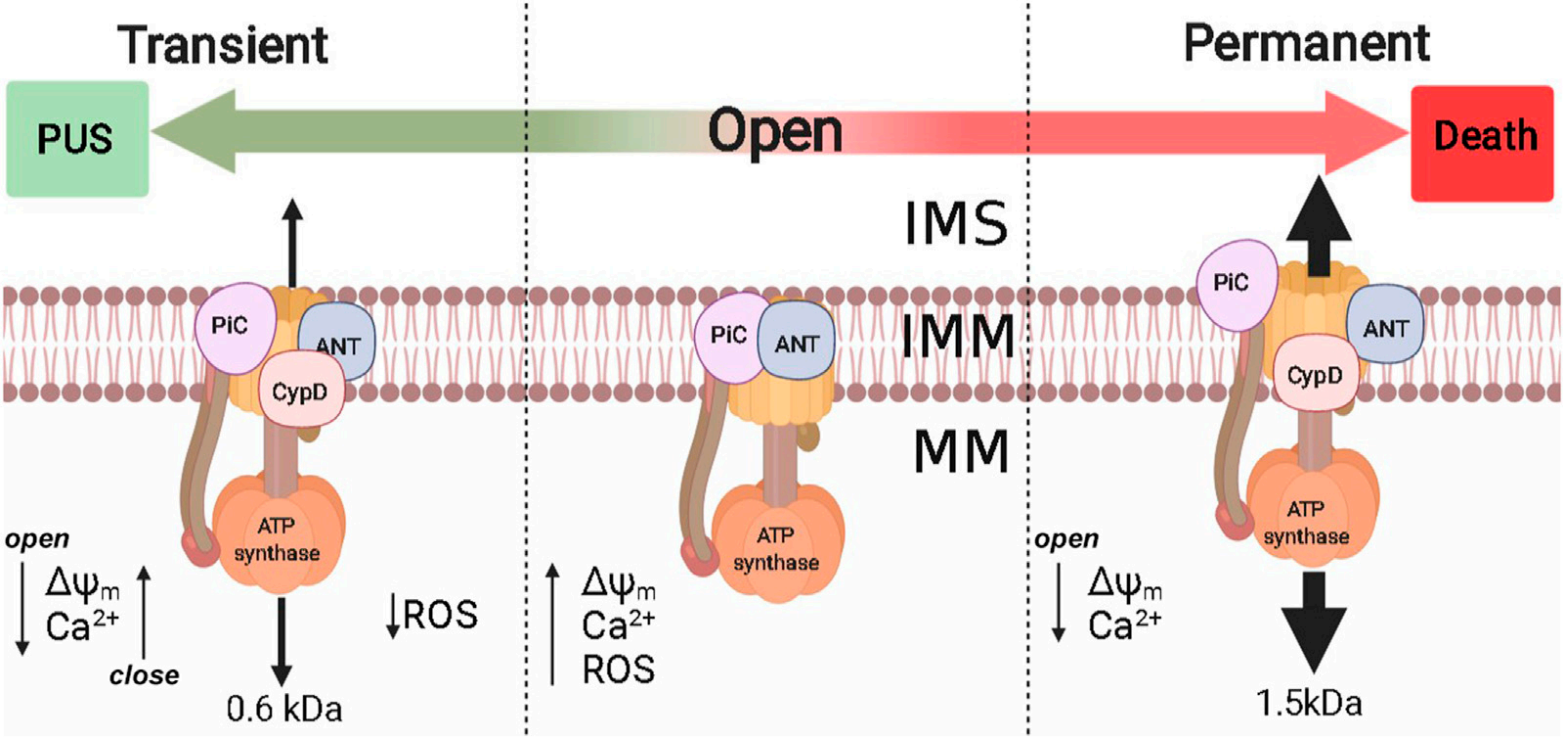
Permeability transition pore and their implications in PUS. Extrinsic PUS mechanisms were developed in eukaryotes and are present from yeast to mammals, probably to control intramitochondrial [Ca^2+^] and ROS production. The molecular identity of PTP is still under debate, although at least Complex V, PiC, ANT, and CypD have been proposed. PTP probably has two opening modes: 1) Transient, where PTP is permeable to molecules up to 600 Da and reversibly decreases ΔΨ_m_ [Ca^2+^]_m,_ decreasing ROS production; 2) Permanent that allows molecules up to 1.5 kDa and metabolite efflux. Nonreversibility may lead to cell death. ΔΨ_m:_ mitochondrial transmembrane potential; [Ca^2+^]_m_, mitochondrial Ca^2+^; IMS, intermembrane space; IMM, inner mitochondrial membrane; MM, mitochondrial matrix.

The second extrinsic OxPhos uncoupling system is the permeability transition pore (PTP) (Haworth and Hunter, 1979). PTP seems to undergo different open states, a transient open state allows passage of up to 600 Da molecules (Boyman et al., 2019) while the fully open state is permeable to larger molecules up to 1,500 Da, which has been proposed for years as a mechanism leading to cell death (Akopova and Sagach, 2005; Palmeira and Rolo, 2012). However, PTP opening reversal has been reported, even in proteoliposomes containing bovine ATP synthase (Urbani et al., 2019). PTP opening reversibility, promotes transient small decreases in mitochondrial ΔΨ. Thus, frequent PTP opening/closing cycles (flickering) would result in matrix Ca^2+^ depletion and increased rate of electron flow that would in turn prevent ROS overproduction. Flickering has been studied mostly in heart, muscle, and astrocytes (Figure 4). In isolated heart mitochondria PTP opening of pore may last 10 min and addition of 1 mM of EGTA can still close the pore and restore ΔΨ (Crompton et al., 1987).

In the rat heart, alternative opening/closing of PTP in a process known as preconditioning decreases mitochondrial Ca^2+^ and prevents ROS overproduction, which prevents tissue damage (Saotome et al., 2009). Another instance where PTP flickering may be helpful for the heart is during extremely demanding activity where O_2_ consumption increases up to ten times (Jafri et al., 2001). The transition of PTP opening from transitory to permanent has been linked to heart failure (Giordano and Giordano, 2005; Belosludtsev et al., 2020). In intact cardiac, striated muscle, kidney cells, and astrocytes, PTP reversibility does lead to alternative ΔΨ decrease/increase and Ca^2+^ release/uptake that in turn lead to decreased ROS as measured by superoxide “flashes” (Li W. et al., 2016; Lu et al., 2016; Boyman et al., 2019; Feng et al., 2019).

Unicellular eukaryotes such as yeast resist wide [O₂] variations much more efficiently than oxyregulators, most of these possess branched respiratory chains, but in addition may express proton sinks. PTP flickering has been observed in these organisms. In *S. cerevisiae*, the Mitochondrial Unspecific Channel (MUC) (Manon et al., 1998) may remain open for a long time and then be closed by high cytoplasmic Ca^2+^ during the cell cycle or when haploid yeast mate, which is an event where high amounts of ATP are required (Iida, et al., 1990a; Iida, et al., 1990b). When the *S. cerevisiae* MUC closes the ΔΨ_m_ increases, mitochondrial swelling is prevented and the control on O_2_ consumption is restored (Cabrera-Orefice et al., 2014; Gutiérrez-Aguilar et al., 2014; Morales-García et al., 2021). A special case is *D. hansenii* where the MUC is special in the sense that it is sensitive to monovalent cations (Cabrera-Orefice et al., 2010). Mitochondria from oxyconformers such as the branchiopod *A. fransciscana* and the shrimp species *Lepidophthalmus louisianensis*, *Crangon crangon*, *Palaemon serratus*, and *L. vannamei* PTP opening is not induced by calcium overloads and these organelles can store high [Ca^+2^]. The absence of a calcium-regulated PTP in crustaceans has led to propose that other PUS such as UCPs and branched respiratory chains are critical for survival during stress (Menze et al., 2005; Holman and Hand, 2009; Rodriguez-Armenta et al., 2021).

## OxPhos Decreases [O_2_] and Thus Prevents ROS Overproduction

Electrons flow down RC following a redox gradient in a transmembrane sensitive process (Quinlan et al., 2013). During high metabolic activity, when ATP is rapidly consumed, proton gradients are consumed, thus enhancing the rate of electron flow through RC (Nicholls and Ferguson, 2013). The observed high rate of O_2_ consumption is traditionally known as known as state-3 respiration and results from a partial, reversible decrease in ΔΨ_m_ and these conditions decrease ROS production similarly to PUS activation (Guerrero-Castillo et al., 2011). In addition, ATP channeling seems to further promote OxPhos optimization and inhibition of ROS production (Korshunov et al., 1997). That is, the physical interaction of the mitochondrial adenine-nucleotide carrier (ANC) with the cytoplasmic hexokinases and/or the intermembrane space creatine-kinase constitutes a metabolon, where the ATP transported by ANC directly enters the active site of the kinase in an exchange for ADP (da-Silva et al., 2004; Meyer et al., 2006; Santiago et al., 2008). In fact, a recent study in bats, naked mole rats and mice demonstrated that hexokinase association with the adenine-nucleotide carrier optimizes OxPhos efficiency, decreasing ROS production and elongating lifespan (Vyssokikh et al., 2020). Extrapolating from these results it has been proposed that both aging, and lifespan may be controlled by exercising, i.e., by activating state 3 systematically to prevent ROS overproduction (Dey et al., 2016; Delaire et al., 2021).

## ROS Detoxification Systems

When all ROS overproduction systems fail, there is still a second line of defense, namely the antioxidant systems that include the enzymes superoxide dismutase (SOD; EC 1.15.1.1), catalase (CAT; EC 1.11.1.6) and the glutathione recycling system made by glutathione peroxidase (GPx; EC 1.11.1.9), glutathione reductase (GR; EC 1.6.4.2), and glutathione S-transferase (GST; 2.5.1.18). In addition, there are molecules, that sequester ROS, such as glutathione, carotenoids, vitamin C, vitamin A, and vitamin E (Li Y. et al., 2016). ROS detoxification systems work at a steady level but are critical during events leading to massive ROS overproduction such as ischemia/reperfusion or hypoxia/reoxygenation.

Antioxidant systems exist in all kingdoms (Ślesak et al., 2016). These are critical for survival of species usually exposed to extremely variable and stressful environments. Some of these are intertidal invertebrates, hypoxia-tolerant fish, and desiccation/freeze-tolerant creatures (Sokolova, 2018). A particular case occurs in *Clostridium acetobutylicum*, a strict anaerobe organism that contains Fe- and Mn-SODs, heme- and Mn-catalases and in addition 1Fe or 2Fe-SORs that reduce the superoxide anion to H_2_O_2_ (Riebe et al., 2009; Morvan et al., 2021).

## Final Considerations

O_2_ and its toxic derivatives are very strong drivers of evolution. All O_2_-exposed organisms are at risk of being damaged, age and eventually die because of the deleterious actions of ROS. Many systems have been designed to counteract toxicity while using the advantages of aerobic metabolism. However, the two most important mechanisms to prevent ROS toxicity evolved in opposite sense, as avoiding O_2_ by exclusion became optimal, branching of RC decreased and eventually disappeared. That is, as oxyregulators optimized O_2_-exclusion, they enhanced their ATP producing efficiency, and slowly lost stress-response ability in their RCs. It remains to be analyzed whether proton sinks those first appeared in eukaryotes have also lost efficiency or not. Substrate channeling as described above is another ROS overproduction prevention system that seems to exist only in eukaryotes. A second line of defense, which was considered only briefly here, are the ROS detoxifying system that play vital roles during crisis such as ischemia/reperfusion and anoxic periods.
